# Taking Care of Friends: The Implementation Evaluation of a Peer-Focused School Program Using First Aid to Reduce Adolescent Risk-Taking and Injury

**DOI:** 10.3390/ijerph182413030

**Published:** 2021-12-10

**Authors:** Lisa Buckley, Mary Sheehan, Kelly Dingli, Bianca Reveruzzi, Veronica Horgan

**Affiliations:** 1 School of Public Health, The University of Queensland, Herston 4006, Australia; 2 Centre for Accident Research and Road Safety-Queensland, Queensland University of Technology, Kelvin Grove 4059, Australia; m.sheehan@qut.edu.au (M.S.); k.dingli@qut.edu.au (K.D.); b.reveruzzi@qut.edu.au (B.R.); v.horgan@qut.edu.au (V.H.)

**Keywords:** intervening, peer protection, protective behaviour, helping, school connectedness, high school, bystander

## Abstract

Injury is a leading cause of adolescent deaths, with risk-taking associated with a sizeable proportion of injuries and many of those risks undertaken in the presence of peers or with peers’ knowledge. Novel ways to promote safety are required and using the peer-relationship may be an important mechanism for prevention. This study reports on the implementation evaluation of the Skills for Preventing Injury in Youth (SPIY) program. SPIY is a high-school program designed to reduce injury by encouraging peers to look out for one another and prevent risk-taking, complemented by developing peer helping and first aid skills as well as school connectedness. 152 students and 12 teachers who delivered SPIY participated in separate 30 min focus groups and reported on students’ understanding of peer protective behaviour and the program implementation (adherence, dose, quality of program delivery, and participant responsiveness). Students reported on many approaches to protecting friends and both students and teachers reported they found the program interesting, interactive, and able to be delivered. Peer protection messages were relevant and acceptable to teachers and students in a risk-taking harm reduction program to reduce adolescent injury.

## 1. Introduction

Injury has a major and preventable impact on the health of young people. In Australia, injury accounts for more deaths than all other causes combined among 12–24 year olds and can leave serious and long term conditions [1]. Adolescent engagement in alcohol use, violence, road-related, and other risks contributes significantly to this problem. In the U.S., nationally representative data from the Youth Risk Behavior Survey shows 24% of high school students reported they were involved in a physical fight [2]. Further, while alcohol use has declined in the past decade, there were still 29% of adolescents who reported use in the past 30 days [3]. Australia similarly shows a decline in adolescent drinking and reports of use in the past week are at 15% of high school students recorded in Australia’s largest national survey of adolescent substance use [4]. While rates of all risk-taking behaviours have decreased in recent decades, injury is still the leading cause of morbidity and mortality among adolescents [1].

During adolescence, friendship groups become an increasingly significant social relationship with an integral role in shaping behaviour, including risk-taking behaviours [5]. Adolescents drink more with friends and experience more motor vehicle crashes with same-age passengers than those who are alone [6,7]. Even in extreme school violence it is estimated that in 75% of cases the plan was known to others [8].

There has been considerable attention over the years to research and interventions to develop adolescents’ resistance to peer pressure to engage in problematic behaviours. However to capitalise on adolescent friends being present and valuing the safety of their friends, an alternative approach to reducing risk-taking behaviour is to support adolescents to actively intervene to prevent harm. Indeed, studies show adolescents are willing to help and intervene with friends and employ multiple strategies to do so [9,10]. Further, regardless of the strategy, adolescents typically report that when they intervene it is effective in changing the behaviour of their friends [11,12]. The Smart and Stoduto [12] study found that about one-third of their adolescent sample intervened to prevent peers’ alcohol and other drug use and that those adolescents were more likely to abstain, to disapprove of drug use, and more likely to have friends who used drugs.

Efforts to reduce harm for adolescents, particularly those delivered in schools, often focus on a single risk behaviour, for example bullying, alcohol use, and distracted driving [13]. Schools have limited curriculum time and thus focusing on messages that span multiple behaviours may be more efficient and accepted by school staff [14] as well as reflect similar underlying risk and protective factors across the behaviours [15]. In a review of sustainability in school-based programs, Herlitz et al. [16] identified facilitators to sustainability included staff identifying positive student outcomes, having the confidence to deliver programs, and adapting program content into routines. Thus suggesting the importance of understanding teachers’ perspectives as well as delivering interventions into the curriculum across multiple risk behaviours.

Yeager, Dahl, and Dweck [17] suggest efficacy with adolescent school-based health programs involves promoting respect for students as well as reflecting key developmental needs, including developing peer social relationships. They suggest programs that empower adolescents and capture their attention and motivation are more likely to demonstrate efficacy in promoting health behaviour. Looking out for friends and protecting them from harm are key concerns for early adolescents [9] and reflects motivation and respect for the issues most relevant for adolescents. Yeager et al. [17] further indicate respect is similarly required in the way content is delivered and suggest delivering messages through discussion and interaction rather than didactic learning. Such findings suggest understanding the adolescents’ perspectives in the way they prefer messages delivered as well as the content of such messages is critical.

The Skills for Preventing Injury in Youth (SPIY) class-based program focused on developing and strengthening adolescents’ use of positive protective and prosocial outreach strategies towards each other linked with first aid training and promoting school connectedness [18]. The SPIY program was implemented into existing curriculum and thus was able to promote interactions between peers as a key strategy for behaviour change. Further, in this program, strengthening school connectedness developed students’ connections with teachers as well as with peers [19]. In a randomised controlled trial, the SPIY program was found to be effective in reducing student violence after 12 months and alcohol use after six months [20].

The SPIY program sought to reduce risk-taking behaviour and subsequent injuries through changing individuals’ beliefs about risk-taking (through targeting change in Ajzen’s [21] Theory of Planned Behavior constructs) as well as increasing supportive relationships with friends and at school. Students undertook eight classroom sessions designed around first aid skill training to promote discussion and supportive activities with peers. These sessions were integrated into the usual health curriculum lessons. Lessons were approximately one hour and followed a format of a brief example scenario of four friends doing something risky and one getting hurt, learning the first aid skills to control the injury, and interactive prevention exercises designed to consider ways in which the friends might have prevented the injury from occurring [22]. Scenarios were based on research to reflect developmentally relevant experiences [23,24]. Prevention exercises used cognitive behavioural techniques to create change. Table 1 provides a summary.

The program included a day-long teacher training session to develop and promote school connectedness as well as train in the methods of delivering SPIY (see Chapman et al. [19]). Approximately one-third of the session was spent on delivering class activities and the remaining time on identifying teachers’ own skills and resources and strategies to promote school connectedness. We included school connectedness in an effort to support individual change and complement social and contextual protection [19]. Where the school environment can include supportive peers and adults, it is associated with more positive adolescent peer behaviour, including protective behaviour [18]. Teachers were also provided with key resources for delivery including, a detailed manual, student workbooks, slides for each lesson, background information, curriculum indicators, and an optional end of term exam.

This study reports on the implementation evaluation of SPIY and in particular how young people understood the key peer protection messages in the program. An implementation evaluation provides further detail than can be obtained from an efficacy evaluation. Efficacy trials provide information about change, whereas an implementation evaluation can provide details to inform dissemination and refinement for target audiences, examining the way a program is delivered in practice [20]. An implementation evaluation may thus focus on the point of view of those who experience the program, in this case teachers and students. Wertz [25] suggests qualitative research might be used to capture the context of the situation and ‘life-world’ experience and describe the importance of the individuals’ meaning and structure of the topic and thus “informs us of what something essentially is” for the individual [25], p. 168.

This implementation evaluation has been undertaken to provide details to inform dissemination and refinement for target audiences [26]. Dusenbery, et al. [27] provide a framework for assessing the implementation of school-based programs and this was considered from the outset. They suggest, covering assessment of adherence (alignment with program content/goals), dose (material delivered), quality of program delivery (interactivity), and participant responsiveness (program satisfaction). With this study, we aim to provide an implementation evaluation of the SPIY program and, in particular, describe the way peer protection messages are received.

## 2. Materials and Methods

### 2.1. Participants and Procedure

A convenience selection of six intervention schools from the efficacy trial [15] participated in the implementation evaluation, including their students (*n* = 152, 68% male) and teachers (*n* = 12, 80% male). Four schools were state-funded and co-educational and two were private schools (one male-only and one female-only school). The Index of Community Socio-Educational Advantage (ICSEA) indicated that the schools included two in the bottom and two in the top quartile of advantage in the country. ICSEA is a measure of advantage of schools incorporating parental income, employment, education, and occupation data [28].

All grade nine students at participating schools undertook the SPIY program delivered by their teachers as part of the usual health curriculum class lessons. No identifying information except sex was collected from focus group participants. Students in the schools that implemented SPIY had a mean age of 13.46 years just prior to the implementation of SPIY, 85.1% identified their country of birth to be Australia and 3.7% identified that they were Australian Aboriginal and/or Torres Strait Islander peoples. Further 82.2% identified living in a two-parent household.

Focus groups with students and teachers were conducted by trained facilitators in small groups (*n* < 5) within approximately six weeks after the program concluded. All facilitators were trained in psychology and audio-recorded face-to-face discussions that lasted approximately 30 min.

As part of the efficacy trial survey undertaken at the six-month follow-up with the full SPIY cohort, students completed implementation evaluation questions (*n* = 1155, 56% female). Primarily survey questions related to the assessment of efficacy and potentially moderating factors see [20]. Only the implementation questions are described here. In the survey, students reported on their recollection of content with eight yes/no items (e.g., Did they remember the lesson with a creek story?) and a question on their perceived change over time on key program content, change in risk-taking behaviour and peer protection (10 point Likert-type scale). These questions were developed for the purpose of the current study to reflect consideration of ‘dose’ and ‘adherence’ as considered by Dusenbury et al. [27] and to reflect the content of SPIY. Dusenbury et al. [27] reviewed previous school-based program implementation and identified previous studies that used self-reports to assess these constructs. This survey was undertaken in students’ usual classes (45 min) and was managed by researchers (see Buckley et al. [20]). We did not have access to class attendance figures.

### 2.2. Ethical Considerations

Approval for this study was obtained from Queensland University of Technology (approval number: 1100000744) and the relevant education departments (including the state education department, approval number, 550/27/1155) and schools. Active consent to participate was provided from parents at state-funded schools and passive consent (that is, parents returned a signed document only if they did not wish their child to take part) for the two privately-funded schools. Teachers and students provided their own written informed consent.

### 2.3. Implementation Evaluation Measures

In the focus groups a semi-structured script was used to examine the implementation using the Dusenbury et al. [27] framework with deviations for clarification and elaboration from participants. Examples of open-ended questions for students include, “*What did you like best about health [the SPIY program] last term? Why?*”; “*What were the main things you learnt from all your health classes last term?*”; “*Do you think the health lessons helped you with your friends? How?*”; and “*Do you think it’s changed how you look after your mates? Why/why not?*”. Such questions were sometimes followed by general prompts to clarify an alternate perspective, for example if the first responses were positive or negative, address the alternative perspective and to clarify the perspective of students who didn’t initially respond, for example, “*What have you* (*guys) to add*?”, “*Are there things you didn’t like*?”*,* “*What did you think of*…”, ”*Was there anything that could be improved on? What were the things you didn*’*t like?”*

Teacher prompt examples include, “*Overall, what did you like best about the SPIY program*? *Why*?”; “*What did you NOT like about the SPIY program–what needs to be improved? Why?*”; “*How could we have made the program even more interactive?*”; and “*How would you feel about giving the lessons to future year nine students*?”

### 2.4. Data Analysis

Teacher and student transcripts were analysed separately and thematically by following the implementation framework themes of adherence, dose, quality of program delivery, and participant responsiveness [27]. In addition, we identified themes around experience of peer protective behaviour from students. We were focused on our participants’ descriptions of their experience and the meaning that they attribute to their class experience and health behaviour. Through the described focus group process, we attempt to provide participants with a “*descriptive task*…*that specify a focus*”, in our case, around issues of implementation and also to allow for being open to “*content that the participant offers*.” [25], p. 171. 

We developed our themes by first identifying the category and then, where relevant, adding sub-themes. These were also checked across the focus groups for dependability and confirmability (separately for teachers and students). Dependability reflecting consistency in responses across focus groups and confirmability reflected in having multiple researchers independently facilitate and review focus group data [29]. This was undertaken by two researchers and then discussed. Our definitions of themes were flexible and reflected how the participants described each of the implementation areas see [30]. We provide example quotes from teachers and students, noting the gender also, for example, a male teacher is coded as M, Tchr and female student as F, St.

## 3. Results

The implementation evaluation considered student and teacher perceptions of content, adherence, dose, quality of delivery, and program satisfaction (see Table 2 for a summary of findings regarding implementation). We also examined later experiences of peer protection behaviour reported by students and their response to first aid training.

### 3.1. Program Content

Students described features of the program content, including their experiences and perspectives about the peer protection messages. They described the following issues as relevant:

“*You have to look out for your friends and make sure they don’t do stupid things, cause yeah they get hurt and stuff*”.

(F, St)

Students reflected on the content:

“*We covered (being) a good mate*”.

(F, St)

There were, however, students who didn’t identify adherence to the peer protection content, for example:

“*We didn’t do too many of like ‘help your friends’ type thing*”.

(M, St)

Students also reflected on how that content had changed their behaviour:

“*Like if you’re gonna do it, even though it’s unsafe, but then like you have a plan*”.

(M, St)

Their discussion also included different elements to what intervening behaviour looked like, including telling them to stop:

“*umm … Tell them not to do stupid things. Like if they’re about to do something*” (M, St); “*tell them if you wouldn’t do it, then they probably shouldn’t*”;

(M, St)

and

“*Yeah just that generally look out for them and if they (are) gonna do something stupid just stop them, or tell them not to*”.

(M, St)

It also reflected communication around pressure:

“*not pressure them into doing anything*”;

(M, St)

“*try to, reason them out of doing really stupid things*”;

(M, St)

and for some being proactive,

“*by avoiding risky situations you take a lot of, fair few people out of a lot of danger*”.

(M, St)

Students described conditions that would facilitate intervening, including increasing age, being a close friend, parental responses, teacher responses, and peer responses. For example:

“*with age comes responsibility*”;

(M, St)

“*depending like if they’re like your friend and kinda depending how close you are to them*”;

(F, St)

and

“*if my friend’s like in trouble … I’m allowed to stand up for them, sort of thing, like, and I won’t get in trouble by my parents ‘cause they think that that’s alright*”.

(F, St)

However, parental approval was not uniform:

“*if there was danger there my parents wouldn’t want me to do anything*”.

(F, St)

In addition, there appeared to be teacher and peer expectations to protect friends as two students described:

“*like if people in your class kinda like … gotta help them sort of thing, I think that’s what they expect, cause that’s what some teachers told me*”;

(F, St)

and

“*I think there are a lot though they (fellow students) don’t really talk about it they just sort of assume you’d help cause it’s like the good thing*”.

(F, St)

Students also reflected on the barriers to intervening, including peer pressure to engage in risky behaviour, for example:

“*and then there’s also peer pressure, so like if one person is really popular does all these things doesn’t want to listen to it, then what do you think the rest are gonna do? So they’re not gonna listen what-so-ever*”;

(M, St)

In addition, the importance of being a good friend:

“*well if it was someone that you weren’t as good friends with they might not put their trust in you so they might not believe you and if you tell one of your good friends not to do it they’ll actually believe you*”.

(M, St)

Students reported that the program had made them feel better about helping:

“*Yeah like before this I’d be like yeah it’s your choice you can do it if you want, now I’d be like giving them advice*”.

(F, St)

Finally, there were students who indicated that they would not intervene:

“*yeah, but like people can look after themselves*”.

(M, St)

Interestingly, a fellow student then responded:

“*well yeah, but if like someone’s like all messed up then you don’t wanna like muck them up even more*”.

(M. St)

### 3.2. Program Adherence

In the focus groups we asked the students what they did in class and the teachers what they delivered. Content appeared to be covered with understanding risk, first aid and peer protection, as students noted:

“*we had to explain the dangers and stuff*”;

(F, St)

“*it taught us all the skills…like how someone gets hurt*”;

(M, St)

and

“*it’s good to be prepared*”.

(M, St)

They also acknowledged that they learnt:

“*there’s always a consequence and don’t let* (*mates) be irresponsible*”.

(M, St)

Key process areas were also covered:

“*we acted stuff out*”.

(F, St)

Teachers noted potential change among their students in the areas that were covered in the course,

“*knowing who to call and in their own safety and that sort of thing will work…and they’ll retain that information I think*”;

(F, Tchr)

and another noting,

“*I think it gives them the skills to hopefully manage their decision better and not be so completely impulsive about the whole thing*”.

(M, Tchr)

In addition, we asked students about any components of the program they talked about with others outside of class. Some students (not all), usually at least one or two in each focus group identified some discussion with their parents of the key material, for example:

“*Yeah I told them* (*my parents) that today we did CPR on the dummies*.”;

(F, St)

“*I talked about it with my mum*”.

(M, St)

These findings were supported by the earlier survey, in which there was general acknowledgment of change in reduced risk-taking and protecting friends. The mean rating of their perceived change on such factors was above the middle score (range: 1–very unlikely to 10–very likely), including ‘doing less risky things’ (M = 5.67, S.D. = 2.57), ‘stop a friend doing something risky’ (M = 6.70, S.D. = 2.36), and ‘help a friend who is injured’ (M = 7.35, S.D. = 2.26).

### 3.3. Dose

Students were asked at the six-month data collection point whether they remembered completing activities, including scenarios and first aid components. There were generally high rates of recollection for first aid components, DRSABCD (78%), treating bleeding (75%), and treating burns (75%). A minority (13%) of students in the SPIY schools did not recall any of the stories and 1% did not recall any of the first aid content.

While most students agreed they covered a lot of material, some students, whose behaviour was at times considered disruptive, would not participate fully in the lessons:

“*… and make jokes and say ‘oh, it’s not important, we don’t need to know that* (*program content*)’.”;

(F, St)

and

“*… they’d like occasionally read the story (from student workbook) and laugh and be like ‘we’d never do that’.*”.

(F, St)

### 3.4. Quality of Program Delivery–Interaction

A priority in the quality of the delivery strategy of SPIY was to ensure it was delivered with interactive components and discussion. This was primarily focused on interactions with peers in class to promote and normalise key messages. Students described many opportunities for interaction and provided comment on the quality of such interactions. For example:

“*There were lots of discussions and class discussions*”;

(M, St)

and

“*and that it wasn’t just about in the workbook you just sit there and do all your work, there was some bits you could act out (with peers) and there was some practical stuff, you could work on (first aid)*”.

(M, St)

Teachers also noted that the program was interactive for a wide range of students:

“*it was the most engaged I’ve had low level (literacy) kids, who usually don’t participate*”,

(F, Tchr)

and the design facilitated interaction:

“*Just the structure made it really easy to work through. It was self- explanatory, clear, it was easy for the students, it was easy for the teachers, and you know with the written components as well as the prac … getting up and actually getting involved and doing it and practising and you know role playing stuff was really good so for like my particular class it worked really well, they were really engaged and I was lucky because I went down into a drama room for those classes so I actually had space and actually worked great*”;

(F, Tchr)

The interaction appeared to facilitate both building of decision-making skills and wider student involvement:

“*It’s more to do with the decision-making side of it, like ‘what would you do if you were in his shoes?’ or something like that (we discussed)*”;

(M, Tchr)

“*If they (delinquent students) were sitting in the corner talking about how silly this is and you should be able to do whatever you want … then I’d be concerned but, in this program, every student was involved*”.

(M, Tchr)

### 3.5. Participant Responsiveness

Overall, students identified elements that were interesting, helpful, and fun, and teachers also recognized this. We identified further key issues of participant responsiveness relative to experiences with the program materials and the interactive scenario-based learning.

Program materials. The program aimed to provide detailed and easy to use resources for teachers to best support adherence to program content and promote interactive delivery. Generally, the material was viewed positively,

“*Yeah I did, I really liked it*”.

(M, Tchr)

This included structure and timing, for example:

“*I was fairly confident we were going to get through them easily enough and so I was able to embellish and add a bit extra and ask a few deviations on the way, so the timing of it I think was pretty fair*”.

(M, Tchr)

Some teachers made deviations to the content, although this typically included bringing in student stories and scenarios,

“*Real interesting I think, I think some of the kids were surprised how, how well they would manage that, or how well they seemed to say they would manage that stuff and what effects they have on their friends and stuff too and that I found that lots and lots of personal stories (with questions, such as,) what about if this happened*”.

(F, Tchr)

Students noted the materials facilitated some interactive delivery, which was helpful for other students with lower literacy skills:

“*some of them did the demonstration*”;

(M, St)

and

“*probably the practical…they (problem students) don’t really like writing so the practical gets through to them a bit more*”.

(M, St)

Some students had suggestions for improvements to their workbooks but were overall positive:

“*Have like extra games*”;

(F, St)

and

“*We could like map out the, our pictures*”.

(M, St)

“*Mine really liked the books, they were really protective, that was mine and they put their names on them*”.

(F, Tchr)

Scenario-based learning. Similarly, there were positive reports of using scenario-based learning or case studies by teachers and students:

“*And even* (the) *scenarios I think were good too*”*…* “*Yeah I think they seemed to really connect*“ *…* “*But to their own experience*”.

(F, Tchr)

Some teachers did adjust and edit the scenarios either by including more or by editing the existing scenarios. These additions were developed either from their own or students’ experiences. Teachers also responded to what they perceived as attempts to make the material relevant. As one male teacher explained:

“*… so we changed the scenario to suit and we started talking about the brother of your friend that you went along with or your own brother of that age or that sort of thing. I don’t think we were meant to stay slavishly to what was in there but to adapt it to suit the situation, and that was fine, coz that got a fair bit of discussion going, too*”.

(M, Tchr)

“*The names that you used, I always noticed, were largely-could go either way, like Sasha is a boy or girl’s name and Jesse is a boy or girl’s name, but just the scenarios themselves are things that boys would be more likely to partake in, and I know that’s hard because boys are more risk takers … (it worked) in my all-girl and mixed gender class*”.

(F, Tchr)

First aid training. Students enjoyed the first aid training. There were incidents described by students in the focus groups where they had been able to put their skills into practice:

“*Well it was like on the side of the road, so I wasn’t just gonna leave him there and walk past him, and he was like stuck under his bike so I helped him out*”.

(F, St)

Teachers indicated that there was perhaps change among some students:

“*I think we’ve seen a somewhat decrease (in risk taking), especially looking at the boys on camp*”;

(M, Tchr)

“*Yeah, most definitely (first aid was helpful). One of the major ones is that you’re doing something for somebody else. It’s a little mission that I’m on at the moment, I suppose, in that I think people are far too selfish, that we need to have a greater sense of community*”;

(M, Tchr)

and from students:

“*(We learnt) like you could help them (mates) out or something*”;

(F, St)

“*(We learnt) like the whole CPR things*”;

(M, St)

“*Yeah it was kinda helpful so like, when you know about what can happen and you can try and prevent it*”.

(M, St)

When asked if they felt it was beneficial for all students, their replies indicated it would be particularly beneficial for those who they perceived to be delinquent:

“*… especially the people who don’t listen … because they’re more the sporty types*”;

(M, St)

“*… and the ones that are going to kill themselves (through risk-taking, not suicide)*”.

(F, St)

## 4. Discussion

In the current study we evaluated the implementation of SPIY following the Dusenbury et al. [27] framework using structured focus group discussions with students and teachers. In addition, we looked at how program content was understood to be covered and considered and accepted by students including peer protection and first aid training messages. Overall, findings from the implementation evaluation highlight SPIY content was positively described and that the design supported interaction among students and between students and teachers. An important contribution to these findings was that teachers reported positively on the resources, including the class lesson presentations, the student workbooks (whether electronic or pen-and-paper), and the detailed lesson manual.

The program sought to encourage adolescents’ positive attitude towards looking out for their friends and to actively intervene to prevent injury and risk-taking and to positively value looking out for friends. SPIY included building efficacy and confidence to avoid harm, and a perception that friends expect protective behaviour from their friends. This positive approach was favourably received by participants. The strengths-based peer protection approach appears to have acceptance by school community adolescents.

Overall, students described intervening behaviour with friends, but this was with caveats, including preference towards looking after a close friend as well as circumstances when they were supported by other friends. They noted family and teacher leadership as supportive of intervening. Students provided detail of the nuances of their intervening behaviour around talking to friends and telling them not to do something. It was also telling them about the consequences. Students provided nuanced information around friends’ behaviour that needed monitoring and stressed the need to plan ahead and recognise that avoiding situations could be effective in preventing harm.

The findings reflect other studies on peer intervening that describes intervening with close friends and where there is greater perceived risk of harm [10]. This is also shown with bullying and violence [31] and indeed, it is consistent with the classic bystander intervention model of Latané and Darley [32] who suggest a feeling of responsibility and identified danger are key steps in intervening. However, other studies have found that some adolescents aren’t always willing to intervene with some describing it as, ‘none of their business’ [9]. A key benefit of the present study is that the implementation of a program designed (in part) to promote peer intervening is also described by students and teachers in a way that suggests it is accepted and relevant to the target audience in providing peer protection messages. Further, while the SPIY program focused on peer protective behaviours it did so in the context of other supportive relationships (i.e., school connectedness) and changing one’s own risk-taking behaviour (to effectively model and to stay safe). Finally, the peer protection messages incorporated and built upon competency development with the central focus on the first aid skills a critical component.

Adolescent risk-taking is social [9]. As such, it provides the opportunity for a peer, or more likely a friend, to be physically present or have knowledge that an adolescent is about to engage in risk-taking behaviour. A school-based program also provides a normative space for adolescents to share and process ideas (through interactive learning experiences). During early adolescence there is increasing investment in the friendship relationship [33]. Contact with prosocial peers, for example peers who volunteer or help out their teacher has been shown to predict lower aggression [34] and lower risk of initiation of substance use [35]. In contrast, those who had friends who were risk-takers but they themselves were not, were likely to begin involvement in behaviours such as alcohol and drug use. Friendship quality includes support, closeness, and intimacy [36] that may provide the opportunity to discuss risk-taking and to intervene. Further, adolescent friendship has defining features that typically include, being reciprocated, voluntary, and derived from mutual satisfaction [37,38]. Bergin et al. [39] highlight a key value that adolescents identify as integral to friendships—that friends look out for one another.

### Implications for Future Research and Practice

There are a number of limitations in our research that might reflect key areas for future research. This includes the use of a predetermined implementation evaluation framework, with focus group questions that are reflective and suggestive of aspects of the framework. We asked participants to describe their experience of the classroom and their health behaviours for their context and as such, the application of SPIY to different contexts and generalizability of this approach (see Wertz, [25]) needs further investigation. Future research might also seek to test different implementation frameworks [40]. In addition, we relied on self-report data for our implementation evaluation to understand the nuances and descriptions from the participants. However, to assess dose and adherence more comprehensively, observation methods might reflect a more objective method or triangulation of findings across more data sources [27].

Findings showed that students could provide detail and nuance to their intervening behaviour and thus suggests areas for consideration on what might be included in similar programs that seek to support positive peer behaviour. In line with the suggestion of Yaeger et al. [17], it provides a way to respect students and meet their needs. Our findings further showed that health programs can be implemented in usual class lessons where there is commitment from schools. Thus ideally school systems would support the inclusion of a breadth of health messages in their early adolescent curriculum with messages that can be understood and described by those target early adolescents [17]. The SPIY program had a strengths-based focus, seeking to promote positive relationships and recognize individual and social resources. A key finding relates to the sustainability challenge for similar programs to develop ways to support highly valued workbooks and teacher resources.

## 5. Conclusions

Adolescent engagement in risk-taking behaviours is an important health issue. Increasing independence is a normal aspect of adolescent development [30]; but it also brings increased risk exposure and vulnerability. The negative influences of peers in such situations has been extensively studied and strengthening resistance to peer pressures has been given considerable attention. The present study provides research on the potential for friends to have a positive effect in terms of reducing risk-taking and how key experiences and key adults in students’ lives can promote positive intervening behaviour. The research in this paper focuses on the way in which teachers and students describe their acceptance of a program that teaches a positive role for friends, through intervening to reduce the likelihood of risk-taking behaviour and learning first aid skills to lessen associated injury. The research indicates that such programs can be implemented into the school system and that students and teachers describe their support for well-resourced materials in targeted programs.

## Figures and Tables

**Table 1:** Outline of the Scenario-based learning activity, first aid component, prevention exercise, and peer-delivery strategy.

Lesson	Scenario	First Aid	Prevention Exercise	Peer Interaction Teaching Strategy
1	Introduction	Emergency response, check for danger, response (DRSABC)	Brainstorming consequences, normative behaviours, and peers’ expectations	Peer discussions (whole of class)
2	Water safety	Checking airway, breathing, CPR	Problem solving re friends’ collective and individual decisions	Student role play of first aid exercise (small groups)
3	Water safety	Choking, CPR	Problem solving and support seeking–identifying facilitators and barriers to help seeking in different contexts (with peers and adults)	Continued small group role play and work in pairs to consider support seeking
4	Alcohol and drug use	Choking, CPR	Roles of supportive friends and ‘real’ mates	Peer discussions (whole of class)
5	Bicycle safety	Bleeding control	Cognitive appraisal of friendship, role of friends–planning	Peer discussions (whole of class); ‘Think-pair-share’ exercise
6	Sports, violence/bullying	Fracture management	Practice, role play as a group—recognising each member of the group’s behaviour	Student role play with additional peers providing suggestions and feedback
7	Motorcycle off-road injury	Safety, burns, shock	Cognitive restructuring—challenge all-or-nothing thinking about friends’ expectations	‘Think-pair-share’ exercise
8	Passenger injury	Head and spinal management	Problem solving—weighing costs vs. benefits of supportive actions	Peer discussions (whole of class)

**Table 2:** Summary of implementation considerations.

Issues	Example Quotes
Adherence	
Understand risk	(We talked about) and don’t take lifts with the drunk guy (M, St) Yeah, basically, but like I said, I myself don’t put myself in those situations to take such stupid risks, so... (M, St)
First aid	I told them (my parents) that today we did CPR on the dummies (F, St) (We learnt) like you could help them (mates) out or something (F, St)
Peer protection	Yeah like before this I’d be like yeah it’s your choice you can do it if you want now I’d be like giving them advice (F, St) Real interesting I think, I think some of the kids were surprised how, how well they would manage that, or how well they seemed to say they would manage that stuff and what effects they have on their friends and stuff too (F, Tchr)
Dose	
Participate fully	If they (delinquent students) were sitting in the corner talking about how silly this is and you should be able to do whatever you want…then I’d be concerned but in this program, every student was involved. (M, Tchr) Well if they’re silly enough to drink in the first place and then drive, I don’t think that the lessons would appeal to them (M, St)
Quality of program delivery/Interactive	
Peer-focused	There were lots of discussions and class discussions (M, St) I found that to get them on task I really just had to link it, like just story-wise, get them to tell their own story (M, Tchr)
Opportunities	It’s more to do with the decision-making side of it, like ‘what would you do if you were in his shoes?’ or something like that (we discussed). (M, Tchr) Puts you in perspective (M, St)
Participant responsiveness	
Interesting	For me it was just interesting questions (M, St) Because it is, it’s interesting stuff, its stuff that people need to know so (F, St)
Helpful	DRSABCD, that was helpful (M, St) Yeah it was kinda helpful so like, when you know about what can happen and you can try and prevent it (M, St) Yeah, most definitely (first aid was helpful). One of the major ones is that you’re doing something for somebody else. It’s a little mission that I’m on at the moment, I suppose, in that I think people are far too selfish, that we need to have a greater sense of community (M, Tchr).
Fun	We talked about how we used the dummies ‘cause that was probably the funnest part (M, St). Yeah, I think that was probably the funnest part, the practical skills (M, St)’ Sometimes it could be a bit boring (F, St). Just like the writing down (F, St). Maybe you should put some of the games (F, St)
Easy to implement	Like the kids actually enjoyed going, yep talk about it, and then there was you know there was always reflection and going back which sort of helped (F, Tchr) Have like extra games (F, St) and we could like map out the, our pictures (M, St) Mine really liked the books, they were really protective, that was mine and they put their names on them (F, Tchr)

## Data Availability

Data are not publicly available as permission was not obtained from participants to do so.
